# Effects of a mandatory DRG payment system in South Korea: Analysis of multi-year nationwide hospital claims data

**DOI:** 10.1186/s12913-019-4650-8

**Published:** 2019-10-30

**Authors:** Jae Woo Choi, Seung-Ju Kim, Hye-Ki Park, Sung-In Jang, Tae Hyun Kim, Eun-Cheol Park

**Affiliations:** 1 College of Pharmacy, Yonsei Institute of Pharmaceutical Sciences, Yonsei University, Incheon, Korea; 2 Institute of Health Services Research, Yonsei University College of Medicine, 50-1 Yonsei-ro, Seodaemun-gu, Seoul, Korea; 3 Department of Nursing, College of Nursing, Eulji University, Seongnam, South Korea; 4 Department of International Cooperation, Health Insurance Review & Assessment Service, Wonju, Korea; 5 Department of Preventive Medicine, Yonsei University College of Medicine, 50-1 Yonsei-ro, Seodaemun-gu, Seoul, Korea; 6 Department of Hospital Administration, Graduate School of Public Health, Yonsei University, Seoul, Korea

**Keywords:** Mandatory DRG policy, Effect evaluation, Difference-in-difference methodology

## Abstract

**Background:**

In 2002, a voluntary diagnosis-related groups (DRGs) payment system was introduced in South Korea for seven disease groups, and participation in the DRGs was mandated for all hospitals beginning in 2013. The primary aim of this study was to compare results reflective of patient care between voluntary participation hospitals (VPHs) and mandatory participation hospitals (MPHs) governed by either the DRGs or fee-for-service (FFS) payment system.

**Methods:**

We collected DRGs and FFS inpatient records (n=3,038,006) from the Health Insurance Review and Assessment for the period of July 2011 to July 2014 and compared length-of-stay, total medical costs, shifting services to an outpatient setting, and readmission rates according to payment system, time of DRGs implementation, and hospital type. We analyzed the effects of mandatory introduction in DRGs payment system on results for patient care and used generalized estimating equations with difference-in-difference methodology.

**Results:**

Most notably, patients at MPHs had significantly shorter LOS and lower readmission rates than VPH patients after mandatory introduction of the DRGs. Shifting services to an outpatient setting was similar between the groups.

**Conclusions:**

Our findings suggest that the DRGs payment policy in Korea has decreased LOS and readmission rates. These findings support the continued implementation and enlargement of the DRGs payment system for other diseases in South Korea, given its potential for curbing unnecessary resource usage encouraged by FFS. If the Korean government deliberates on expansion of the DRGs to include other diseases with higher rates of complications, policymakers need to monitor deterioration of health care quality caused by fixed pricing.

## Background

Diagnosis-related groups (DRGs) refer to groups of similar patients for consumption patterns of resources and clinical characteristics. DRGs represent a flat per discharge payment that differs based on severity, procedures, and diagnosis [1]. The DRG payment system, as a policy tool for efficiency improvement and cost containment, shifts economic responsibilities from insurers to medical institutions and promotes consciousness for costs among service providers. Thus, medical institutions tend to regulate the payment mechanism by altering medical services [2]. DRG payment systems were first developed at Yale University as an alternative to reimbursement payment system to manage medical expenses. The US government decided to implement to the Medicare program [3, 4]. Numerous Europe countries have also introduced the DRGs payment system to manage medical expenditures and improve efficiency [5].

South Korea started to introduce national health insurance in 1977 and adopted a fee-for service (FFS) system for health care services [6]. FFS refers to a payment system that healthcare provider is paid for each service conducted, and research has indicated that FFS systems result in rapid increases in medical costs [7]. The Korean government decided to introduce a DRGs payment system to solve problems stemming from overtreatment under the FFS system. The Korean government preliminarily adopted a pilot program of the DRGs payment system that reflected national average treatment charges among patients in particular disease groups from 1997 to 2002. Following the pilot stage, Korea implemented a voluntary DRGs payment system for groups of seven disease and gained participation from about 61% of all hospitals nationwide [8]. As of 2012, the government executed mandatory participation in the DRGs system, beginning with relatively small medical institutions (clinics or hospitals), followed by larger hospitals (general hospitals or tertiary hospitals) in 2013 [9].

For South Korea, previous literatures have studied the effect of implementation of the DRGs policy and have often identified significant reductions in length-of-stay (LOS) and out-of-pocket payments, compared to the FFS system, despite some results to the contrary [10–15]. The effect of the DRGs system for quality of care has also been controversial [8, 16]. However, these inconsistencies may be explained by the fact that many studies only examined effects in voluntary participation hospitals (VPHs), which likely manage more efficiently than other hospitals. Little is known about the effect of the DRGs payment system utilizing nationwide data from both voluntary and mandatory participation hospitals (MPHs).

Therefore, we examined LOS, total medical cost, shifting services to outpatient settings, and readmission rate for patients from VPHs and MPHs governed by either the DRGs or FFS. We also analyzed the effects of the DRGs policy according to hospital type and date of DRGs implementation.

## Methods

### Data sources and study design

The Health Insurance Review and Assessment (HIRA) dataset includes information on the medical and pharmacy bills for all Koreans. All hospitals or pharmacies turn in claims dataset for outpatient and inpatient care, including demographic information, procedures, diagnoses, and prescriptions, to the HIRA to get reimbursement of medical expenses from the government. The HIRA data were based on the sixth edition of the Korean Classification of Disease, which is a revised version of the tenth edition of the International Classification of Diseases code [17].

From the database, we extracted inpatient DRGs data, because DRGs only apply to admitted patients. We also excluded records from inpatients who received medical aid, as the DRGs system only includes recipients of national health insurance. We merged the DRGs data with another hospital-based dataset related to patients treated under the FFS system: the dataset was extracted by the HIRA using unique patient IDs. We then collected all information of inpatients from July 2011 to July 2014 with one of seven DRG-approved diseases (KDRGs codes: C051, C052, C053, C054 [cataract surgery], D111 [tonsillectomy and adenoidectomy], G081, G082, G083, G084 [appendectomy], G095, G096, G097, G098 [herniotomy], G102, G104, G015, G106 [hemorrhoidectomy], N041, N042, N045, N046, N047, N048 [hysterectomy], and O016, O017 [cesarean delivery and hysterectomy and adneelomy]). In addition, we included general information about the admitting hospital, such as ownership, type, teaching status, location, and numbers of doctors, nurses, and beds, in our dataset. This study received ethical approval for this research from the institutional review board of the Yonsei University Graduate School of Public Health.

### Study sample

We included records of DRGs claim data and FFS claim data and grouped them according to the date DRGs was implemented. Hospitals were classified as clinics; hospitals providing primary care, including inpatient services; general hospitals; and tertiary hospitals with more than 100 beds [18, 19]. In multivariate analysis, we combined hospital types as large hospitals (general hospitals + tertiary hospitals) and small hospitals (clinics + hospitals).

### Dependent variables

This study explored four items reflective of patient care (LOS, total medical costs, shifting services to outpatient settings, and readmission rates). These items have often been utilized in studies of DRG effects in Korea [15, 20–23]. We assessed LOS using admission date and discharge date. This research estimated total medical costs as the sum of FFS or DRGs claims for each inpatients, and we utilized each year’s increasing rate of negotiated medical price to adjust medical expenses to 2014 levels. Shifting services to an outpatient setting was defined as visiting outpatient institutions within 14 days before or after hospitalization based on the admission date or discharge date, respectively. We defined readmission rate as readmission with same primary diagnosis within 30 days after discharge at the same or another hospital. This study excluded results for cataract surgeries in the readmission analysis because hospitals claimed the same disease code when a doctor operated on the patient’s other eye before mandatory participation of the DRGs system. This type of readmission differed from our definition, which depended on the readmission being clinically related to a prior admission [24].

### Independent variables

**Fig. 1:**
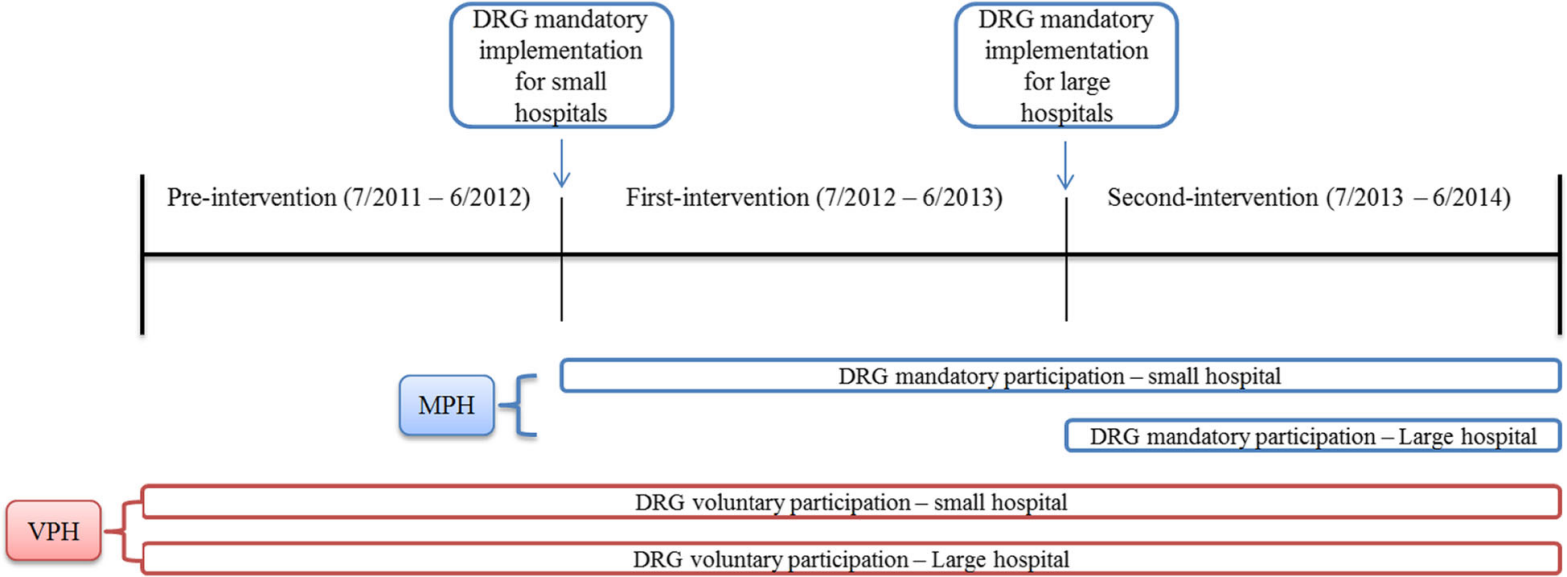
Timeline for study subjects and study period

This study compared the results on patient care in MPHs and VPHs. An MPH was defined as a hospital that continued with the FFS system until July 2012 (clinics and hospitals) or July 2013 (general and tertiary hospitals), whereas VPHs comprised hospitals that implemented the DRGs system at least 6 months before the mandatory participation of the DRGs system. Hospitals that moved to DRGs payment within 6 months before mandatory participation of the DRGs system were excluded in this study. Thus, the experimental group comprised patients who received care at MPHs, and the comparison group included patients treated at VPHs (Fig. 1).

### Covariates

We matched patient level data to the hospital information at which the patient had been admitted. Patient level data included sex (female, male), age group (20-34, 35-49, 50-64, 65-74, 75+ years), and Charlson comorbidity index (CCI; 0, 1, 2, 3, 4+), which is constructed based on ICD-9 codes. The Charlson comorbidity index is the sum of weighted scores allocated to main health conditions [25].

Hospital-level data included type (clinics, hospitals, general hospital, and tertiary hospital), ownership (private, public), teaching status (teaching, non-teaching), sizes (number of beds), human resources (number of doctors or nurses), and locations (urban or rural).

### Statistical analysis

This study calculated summary statistics by calculating means and standard deviations for and frequencies and percentages using chi-square test and t-test. Chi-square tests of associations were utilized to assess differences in proportions by mandatory participation in the DRGs system.

This research used multivariate models to evaluate the effect of mandatory participation in DRGs on LOS, total medical cost, shifting services to outpatient settings, and readmission. This study adjusted the models for principal diagnosis and patient- and characteristics in hospital-level and then performed generalized estimating equations (GEE). This study also utilized logit-link GEE to assess shifting services to outpatient settings and 30-day readmission and identity-link normal-distribution GEE to evaluate log-transformed total medical cost and LOS.

This study utilized a difference-in-differences methodology to compare pre- and post-reform changes between MPHs and VPHs, in which we controlled for baseline differences between the hospital groups. Previous studies have used the difference-in-differences approach to estimate the effect of policy alteration or introduction of new system [26, 27]. The difference-in-differences methodology is a standard policy assessment tool that explores the independent effect of introduction of policy on a case group in comparison with a control group once any policy is implemented. The case group of this study comprised patients admitted to hospitals that mandatorily participated in the DRGs system, while the control group consisted of those who admitted to hospitals that voluntarily participated in the DRGs system. Using data from patients before the mandatory implementation of DRGs, we were also able to control for pre-reform trend differences to ensure that the estimated impacts were not biased due to different baselines for MPHs and VPHs. The DID estimator can be calculated only if in pre-treatment period parallel trend assumption hold. We tested the parallel trend assumption by hospital sizes (clinics and hospital; general and tertiary hospital) and the parallel trend assumption was satisfied (Additional file 1: Figure S1 and S2).

## Results

We collected records from 3,038,006 patients (DRGs claim data: 2,565,902; FFS claim data: 472,104) and grouped them according to date of DRGs policy implementation (pre- intervention: 1,011,057, July 2011 to June 2012; first intervention: 1,014,627, July 2012 to June 2013; or second intervention: 1,012,322, July 2013 to June 2014). We collected records for adults who were discharged with a principal diagnosis for cataract surgery, tonsillectomy and adenoidectomy, appendectomy, herniotomy, hemorrhoidectomy, hysterectomy, or cesarean delivery from one of 3,626 hospitals between July 2011 and July 2014. Of these patients, 28.8% were cared for by 1,432 MPHs, and 71.2% were cared for by 2,194 VPHs. The mean age of the patients at MPHs was 45.4 years, and 71.4% were women. Cesarean delivery accounted for 25.7% of MPH cases, and approximately half of the patients demonstrated a CCI of 0. The mean age of VPH patients was 54.1 years, and 60.6% were women. Cataract surgery accounted for 47.5% of these cases, and 29.1% of the patients had a CCI of 0.

Table 1 shows summary statistics of patient and hospital characteristics in this study. At the hospital level, more than half of MPHs were clinics, and approximately 80% were private medical institutions that depended almost exclusively on payments for their revenue. Over 86% of MPHs served patients who lived in urban. The average numbers of physicians, nurses, and beds were 29, 54, and 118, respectively. Approximately 85% of VPHs were clinics, and 93.6% were private hospitals. Over 90% served patients who lived in urban, and the average numbers of VPH physicians, nurses, and beds were 5, 9, and 30 respectively.

**Table 1:** General characteristics of patients and hospitals in this study

Variables		Total	Patients in MPHs (N: 877,012)		Patients in VPHs (N: 2,160,994)		*p*-value
			N	%	N	%	
Patient characteristics							
Age							<.001
	20-34	771,461	308,009	35.1	463,452	21.5	
	35-49	706,019	260,116	29.7	445,903	20.6	
	50-64	584,997	148,509	16.9	436,488	20.2	
	65-74	585,253	97,911	11.2	487,342	22.6	
	75-	390,276	62,467	7.1	327,809	15.2	
	Mean ± SD - yr	52.3±7.1	45.4	±6.4	54.1	±8.7	<.001
Sex							<.001
	Male	1,102,541	251,154	28.6	851,387	39.4	
	Female	1,935,465	625,858	71.4	1,309,607	60.6	
Principal diagnosis							<.001
	Cataract surgery	1,191,471	164,350	18.7	1,027,121	47.5	
	Tonsillectomy & adenoidectomy	34,108	29,514	3.4	4,594	0.2	
	Appendectomy	189,782	153,249	17.5	36,533	1.7	
	Herniotomy	62,042	40,962	4.7	21,080	1.0	
	Hemorrhoidectomy	793,650	81,239	9.3	712,411	33.0	
	Hysterectomy	279,737	182,766	20.8	96,971	4.5	
	Cesarean delivery	487,216	224,932	25.7	262,284	12.1	
Charlson comorbidity index (CCI)							<.001
	0	1,029,110	401,308	45.7	627,802	29.1	
	1	476,180	168,393	19.2	307,787	14.2	
	2	377,376	99,015	11.3	278,361	12.9	
	3	484,961	89,543	10.2	395,418	18.3	
	≥4	670,379	118,753	13.5	551,626	25.5	
Hospital characteristics							
Hospital type							<.001
	Tertiary hospital	44	44	3.1	0	0	
	General hospital	294	198	13.8	96	4.4	
	Hospital	582	346	24.2	236	10.8	
	Clinic	2,706	844	58.9	1,862	84.9	
Hospital ownership							<.001
	Public	36	1	0.1	35	1.6	
	Corporation	405	299	20.9	106	4.8	
	Private	3,185	1,132	79.1	2,053	93.6	
Teaching status							<.001
	Teaching	157	122	8.5	35	1.6	
	Non-teaching	3,469	1,310	91.5	2,159	98.4	
Location							<.001
	Urban	3,216	1,237	86.4	1,979	90.2	
	Rural	410	195	13.6	215	9.8	
Number of bed - mean ± SD		148±106	118	±86	30	±21	
Number of doctor - mean ± SD		34±47	29	±46	5	±4	
Number of nurse - mean ± SD		63±72	54	±82	9	±12	

*P*-values were calculated with the use of Chi-square tests (or Student’s t-test) of association unless otherwise indicated

*MPH* mandatory participation hospital

*VPH* voluntary participation hospital

Table 2 shows that the average LOS of patients treated at MPHs and VPHs reduced by 1.15 and 0.1 days, respectively, with regard to the first intervention. At the second intervention, LOS for MPH and VPH patients decreased by 0.76 and 0.25 days, respectively. The average total medical cost for MPH patients increased by $235, whereas that for VPH patients decreased by $27 after the first intervention. However, the total medical cost for VPH patients increased by $41 at large hospitals at the second intervention. Table 3 shows that the shifting of services to outpatient settings among patients cared for at MPHs and VPHs increased by 4.2 (p<.001) and 1.1 (p<.001) percentage points (p.p.) among small hospitals, respectively (first intervention effect), while this increased by 2.1 (p<.001) and 2.2 (p<.001) p.p. among large hospitals (second intervention effect), respectively. Finally, readmission of MPH and VPH patients decreased by 1.1 and 0.1 p.p. among small hospitals (first intervention effect), respectively, and readmission of MPH and VPH patients decreased by 1.2 and 0.3 p.p. among large hospitals (second intervention effect), respectively.

**Table 2:** Regression model estimates for length-of-stay and total medical cost by DRG policy intervention period

Variables	Pre-Intervention	First Intervention			Second Intervention		
	7/2011-6/2012	7/2012-6/2013			7/2013~6/2014		
	Mean±SD	Mean±SD	*B*	DID	Mean±SD	*B*	DID
Length-of-stay							
Large hospital							
MPH	5.37±4.08	5.22±3.93	0.98***	1.01	4.21±2.51	0.86***	0.89***
VPH	5.71±3.27	5.52±2.73	0.97***		5.27±2.45	0.97***	
Small hospital							
MPH	6.49±2.99	5.34±2.49	0.91***	0.93***	4.53±2.65	0.96***	0.97***
VPH	2.55±2.18	2.45±2.06	0.98***		2.35±2.00	0.99***	
Total medical cost							
Large hospital							
MPH	1382±710	1353±705	0.98***	0.87***	1556±675	1.18***	1.15***
VPH	1370±497	1549±568	1.20***		1590±600	1.02***	
Small hospital							
MPH	1032±401	1267±440	1.33***	1.38***	1188±469	1.01***	1.01***
VPH	836±216	809±301	0.95***		802±309	1.00	

*MPH* mandatory participation hospitals

*VPH* voluntary participation hospitals

*DID* difference-in-differences

Large hospital: tertiary hospitals + general hospitals; Small hospital: hospitals+ clinics.

Adjusted odds ratios obtained from generalized estimating equations analysis with all of the variables in Table 1.

All costs were controlled by medical insurance fees, which are annually adjusted and include un-insured costs.

1 USD = 1,200 Korean won (02/2016)

***p<0.001

**Table 3:** Regression model estimates for shifting services to outpatient settings and readmission by DRG policy intervention

Variables	Pre-Intervention	First Intervention			Second Intervention		
	7/2011-6/2012	7/2012-6/2013			7/2013~6/2014		
	N (%)	N (%)	*B*	DID	N (%)	*B*	DID
Shifting services to outpatient settings							
Large hospital							
MPH	55,388(31.12)	64,398(35.43)	1.25***	0.94***	68,360(37.50)	1.09***	1.00
VPH	13,373(28.67)	14,111(33.84)	1.33***		14,247(36.08)	1.10***	
Small hospital							
MPH	12,141(14.29)	20,047(18.46)	1.13***	0.99	31,553(22.31)	1.08***	1.02
VPH	339,579(48.32)	337,344(49.42)	1.05***		321,412(49.61)	0.99*	
Readmission							
Large hospital							
MPH	4,142(2.98)	4,022(2.85)	0.95*	1.14*	2,312(1.67)	0.59***	0.82*
VPH	597(1.42)	433(1.16)	0.85*		300(0.85)	0.73***	
Small hospital							
MPH	1,576(1.86)	702(0.72)	0.38***	0.40***	735(0.66)	0.92	0.99
VPH	3,103(0.86)	2,829(0.82)	0.95		2,548(0.80)	0.96	

*MPH* mandatory participation hospitals

*VPH* voluntary participation hospitals

*DID* difference-in-differences

Large hospital: tertiary hospitals + general hospitals; Small hospital: hospitals+ clinics.

Adjusted odds ratios obtained from generalized estimating equations analysis with all of the variables in Table 1.

Patients with cataract surgery as the primary diagnosis were excluded in total analysis.

*p<0.05 ***p<0.001

After adjusting for principal diagnosis, patient- and hospital-characteristics, we discovered that the average LOS of MPH patients reduced, compared to VPH patients (adjusted odds ratio: 0.93, 0.89; 95% confidence interval [CI], 0.92 to 0.94, 0.88 to 0.90) (Table 3). We observed these LOS trends for all diagnoses except cataract surgery (Additional file 2: Table S1). The total medical expenses of MPH patients increased (adjusted odds ratio: 1.38, 1.15; 95% CI: 1.37 to 1.39, 1.14 to 1.16) except in cases of tonsillectomy and adenoidectomy and hemorrhoidectomy (Additional file 2: Table S2). Table 3 shows that shifting services to outpatient settings for MPH and VPH patients did not differ significantly (adjusted odds ratio: 0.99, 1.00; 95% CI: 0.96 to 1.01, 0.97 to 1.03), although we did observe some disease-specific variations (Additional file 2: Table S3). Finally, this study found that readmission rates of MPH patients reduced (adjusted odds ratio: 0.40, 0.82; 95% CI: 0.36 to 0.45, 0.70 to 0.96); these trends were similar among all diagnoses (Additional file 2: Table S4).

## Discussion

In our broad observational study, we found that patients treated at MPHs had significantly shorter LOSs and lower readmission rates than VPH patients following mandatory participation of the DRGs and that shifting services to outpatient settings was similar between the patient groups. In addition, the total medical costs incurred by MPH patients increased following mandatory participation of the DRGs system.

First, we found that LOS among MPH patients was significantly lower than that among VPH patients following mandatory participation of the DRGs system. This result was similar to previous findings that indicated the average LOS of patients with certain diagnoses declined during the DRG pilot program (1997 to 2000) and implementation of the voluntary DRG system (2004 to 2011) in South Korea [15, 28]. Results for other countries also indicated that a DRGs payment shortened LOS [2, 29]. One of the major purposes of DRGs systems is to reduce LOS, as well as medical expenditures, for the patient. The average LOS in Korea in 2013 was 16.5 days, indicating it the second highest among the Organization for Economic Cooperation and Development behind Japan (Japan: 17.2 days) [30]. The LOS in Korea is increasing annually, while that in Japan has reduced. It is interested that LOS reduced significantly following DRGs implementation despite the surgical procedures utilized by the Korean DRGs system are simple relatively. More importantly, this significant reduction in LOS may be a long-term effect of the system, as we observed reduced LOS during the second intervention period. Insurers need to monitor treatment processes and results because the DRGs payment system may expedite negative actions, such as inappropriate early discharges of patients who had unstable conditions [31].

Second, total medical costs among MPH patients, but not VPH patients, increased following mandatory participation of the DRGs system, and the magnitude of total medical costs reimbursed by the DRGs system is greater than FFS system in Korea. These differences may result in respective price adjustments for individual diseases and insurance benefit approval for non-insured diagnoses. The Korean Hospital Association has contracted with insurers following the mandatory participation of DRGs, and many medical departments have complained strongly that such contracts were finalized without their agreement. In addition, they resisted the DRGs system until insurers reorganized the disease classification system and provided sufficient increases in reimbursements for patient care. As a result, insurers have adjusted their payment rates to encourage medical community participation in and to minimize discontent with the mandatory participation of the DRGs system during the second intervention period. However, we suggest that these price increases may be an inappropriate long-term policy. The government should investigate the original prices for medical practice, materials for medical treatment, and pharmaceuticals, and implement a revised plan with reasonable indemnification. Also, the proportion of un-insured patient costs that Korean hospitals can coordinate arbitrarily, compared to the total medical costs, is high. The large proportion causes a relatively low public health care expenditure of 56% in 2013, which was lower compared to the OECD average (72%) and is the fourth lowest of OECD countries following Mexico (51%), the United States (48%), and Chile (46%) [32]. The government has converted some un-insured expenses to insured costs, and insurers predict decreasing out-of-pocket costs by about 20% with the DRGs system. Therefore, potential increases in total medical expenses will be affected by these conversions of un-insured expenses. We suggest that the government need to examine alternations for stabilizing the application of un-insured costs by expanding the diseases and diagnoses included in the DRGs system.

Third, because the DRGs system reimburses costs for specific diseases based on a fixed price, physicians might shift inpatients to an outpatient service to decrease medical treatment or examination of inpatients. A prior report reported that after participation of the DRG system, the volume for pre-surgery examinations increased prior to hospitalization [33]. However, this study found no evidence that shifting services to outpatient settings increased following mandatory participation of the DRGs system. Although shifting services to outpatient settings for MPH patients increased significantly, a similar observed increase in VPH patients shifting services to outpatient settings suggests that mandatory DRGs participation is not the main cause of these transfers. A previous study that indicated the average number of medical tests conducted prior to admission increased from 3.51 to 4.46 following DRGs participation [28], although we attribute this difference to study design. Before controlling for other factors, we found that the rate of shifting services to outpatient settings increased by approximately 2-4 p.p. at MPHs, which corroborates the above-mentioned study. However, we considered a control group in our study design and believe this approach resulted in a more robust analysis of DRGs policy effects.

Finally, readmission rates, excluding those of patients with cataract surgery, decreased significantly after mandatory implementation of DRGs, indicating an overall improvement in the quality of patient care [34–44]. Our finding differed from previous research in which hospitals discharging patients early had increased readmission rates [45]. This study implied that DRGs payment systems may decrease quality of care because of “under-treatment” of specific conditions. But, there was no evidence that readmission rates decreased following mandatory participation of DRGs. These unexpected, yet positive, results may be due to more accurate medical coding, as insurers instruct all hospitals to submit separate DRGs claims, regardless of FFS claims already made. Our findings related to reduced readmission rates during the second intervention period supports this conclusion. Future research is warranted explore readmission rates of complicated procedures because the seven diseases applied by DRGs systems are treated by relatively simple surgical procedures.

This study has the a few limitations. First, our study did not consider un-insured costs in the total medical expenses. The Korean un-insured costs accounted for 24% of total medical expenditure in 2014. This research also did not explore alterations in out-of-pocket costs in the DRGs system because of our limited dataset. Second, we controlled for hospital characteristics in our regression models, although additional differences between MPHs and VPHs may have influenced our findings. For example, VPHs may have elected to participate in the DRG system because they already operated more efficiently than other hospitals, which could impact results on patient care independently of DRG implementation. Third, because we examined seven diseases, these study findings cannot be generalized to all diseases. Fourth, because our research has utilized the administrative data, our study might have overlooked any complications of the patients. But, we utilized CCI scores of each patient for evaluating the clinical characteristics of patients to resolve this limitation partially. Finally, there is the possibility that patients were first treated in a hospital under FFS and then in a hospital under the DRG system.

## Conclusions

The mandatory participation of the DRGs system has caused significant decreases in LOS and readmission rate without increased shifting services to outpatient settings for patients with one of seven DRG-related diagnoses. Our findings support the continued implementation and enlargement of the DRGs payment system for other diseases in South Korea, given its potential for curbing unnecessary resource usage encouraged by FFS. If the government deliberates on expansion of the DRG system to include other diseases with higher rates of complications, policymakers need to monitor deterioration of health care quality caused by fixed pricing.

## Supplementary information

**Additional file 1: Figure S1.** Parallel trend assumption in case of large hospital. **Figure S2.** Parallel trend assumption in case of small hospital.

**Additional file 2: Table S1.** Regression model estimates for length-of-stay according to DRG policy intervention period and primary diagnosis. **Table S2.** Regression model estimates for total medical cost according to DRG policy intervention period and primary diagnosis. **Table S3.** Regression model estimates for shifting services to outpatient settings according to DRG policy intervention and primary diagnosis. **Table S4.** Regression model estimates for readmission according to DRG policy intervention and primary diagnosis.

## Data Availability

The datasets generated and/or analysed during the current study are not publicly available because the Health Insurance Review and Assessment (HIRA) of Korea requests all related researchers to pledge not to review, share, or release the database. It is possible for other researchers to require access to the dataset directly from the HIRA.

## Abbreviations

DRG: Diagnosis-related groups
VPH: Voluntary Participation Hospitals
MPH: Mandatory Participation Hospitals
FFS: Fee-For-Service
GEE: Generalized estimating equations
LOS: Length-Of-Stay
HIRA: Health Insurance Review and Assessment
OECD: Organization for Economic Cooperation and Development
