# Supplementary material for: Effects of a mandatory DRG payment system in South Korea: Analysis of multi-year nationwide hospital claims data
Source: BMC Health Serv Res. 2019 Oct 30;19:776. doi: 10.1186/s12913-019-4650-8 (PMC6822472; doi:10.1186/s12913-019-4650-8)
Supplement: Supplementary file 1 — Additional file 1: Figure S1. Parallel trend assumption in case of large hospital. Figure S2. Parallel trend assumption in case of small hospital. [file 12913_2019_4650_MOESM1_ESM.doc]

| 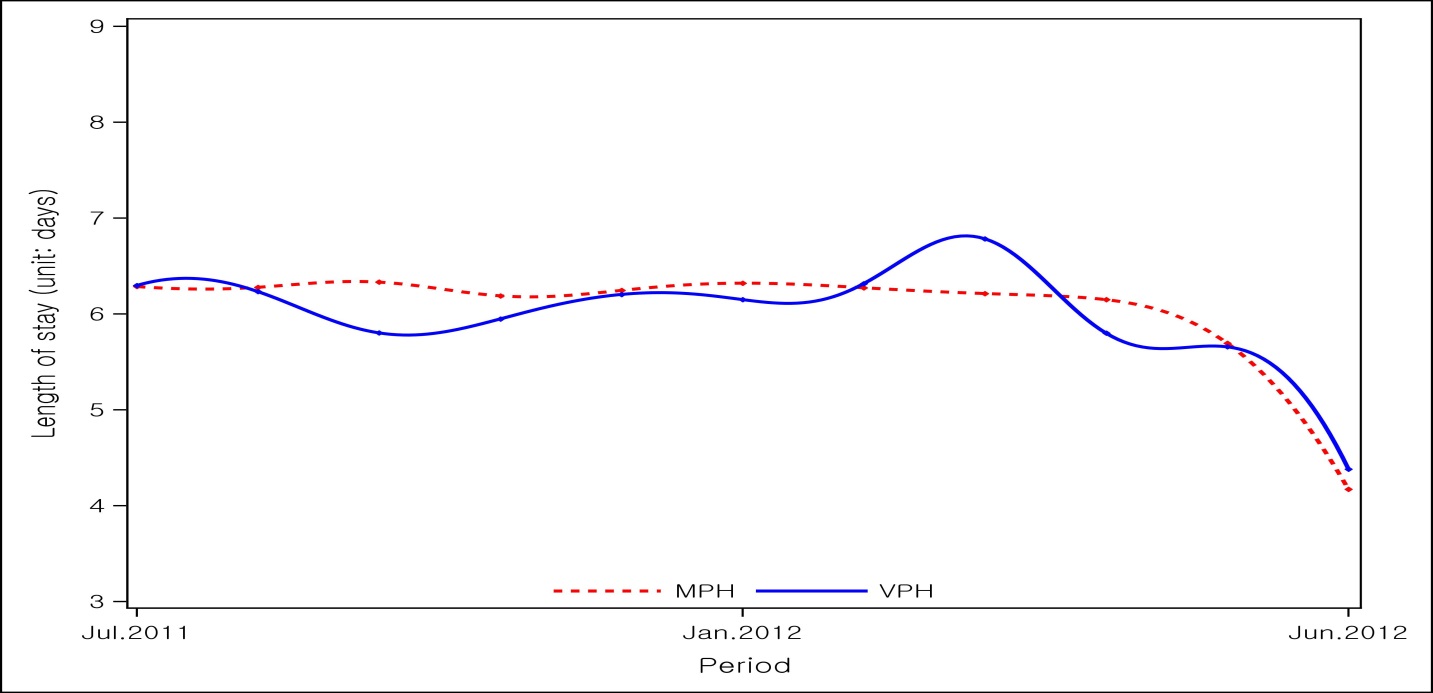 | 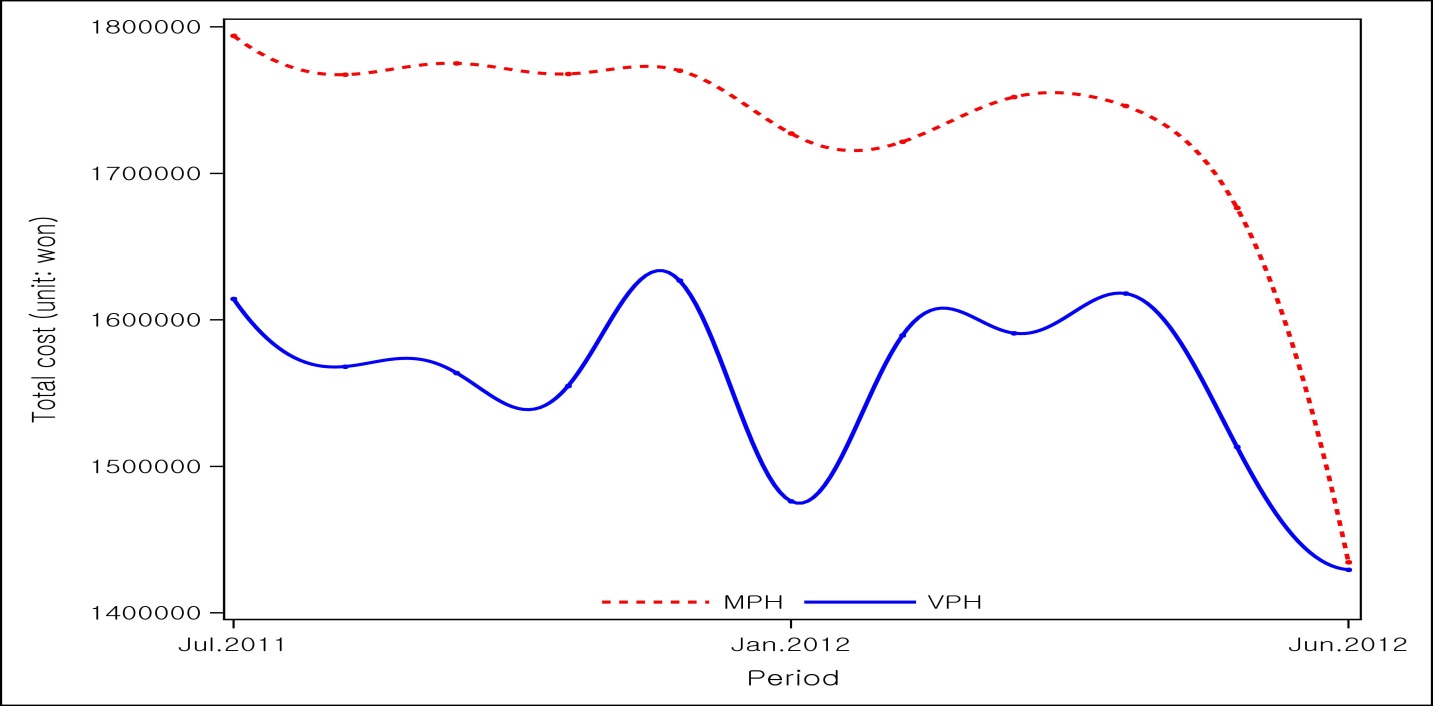 |
| --- | --- |
| (A) Length-of-stay | (B) Total medical costs |
| 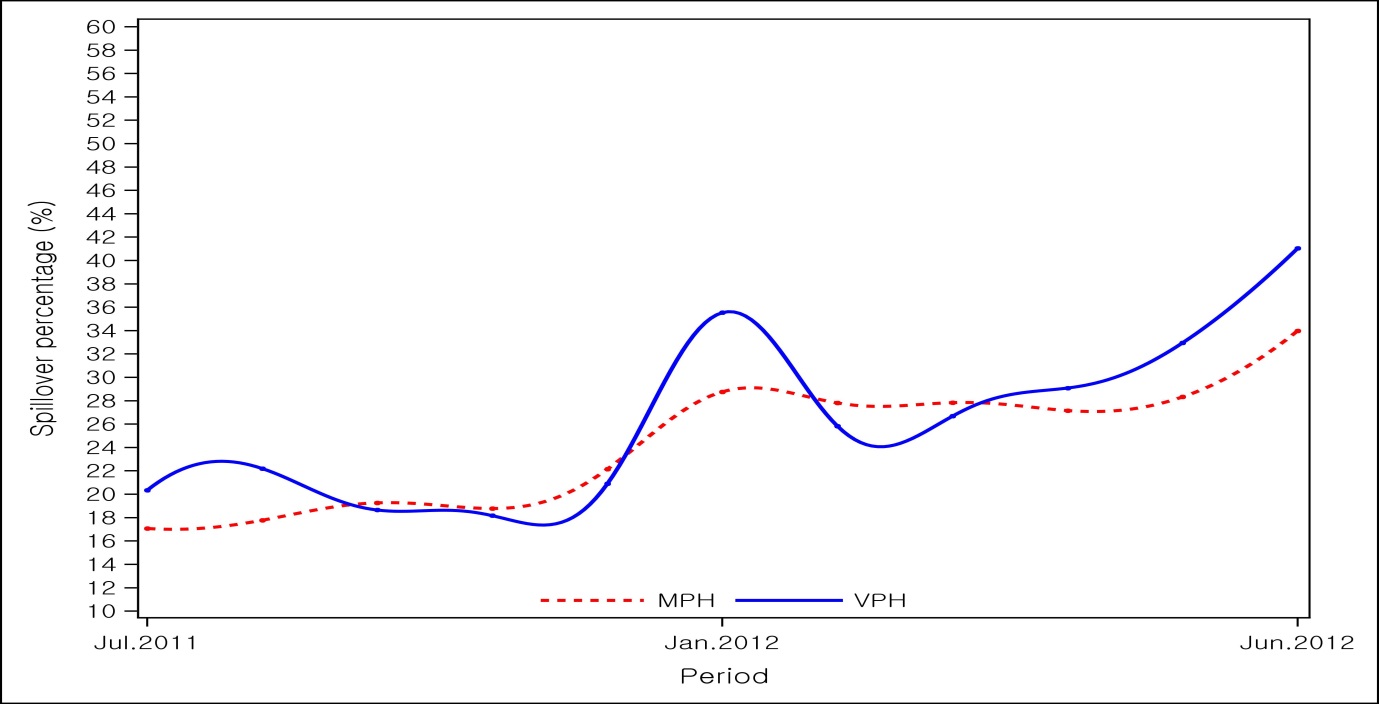 | 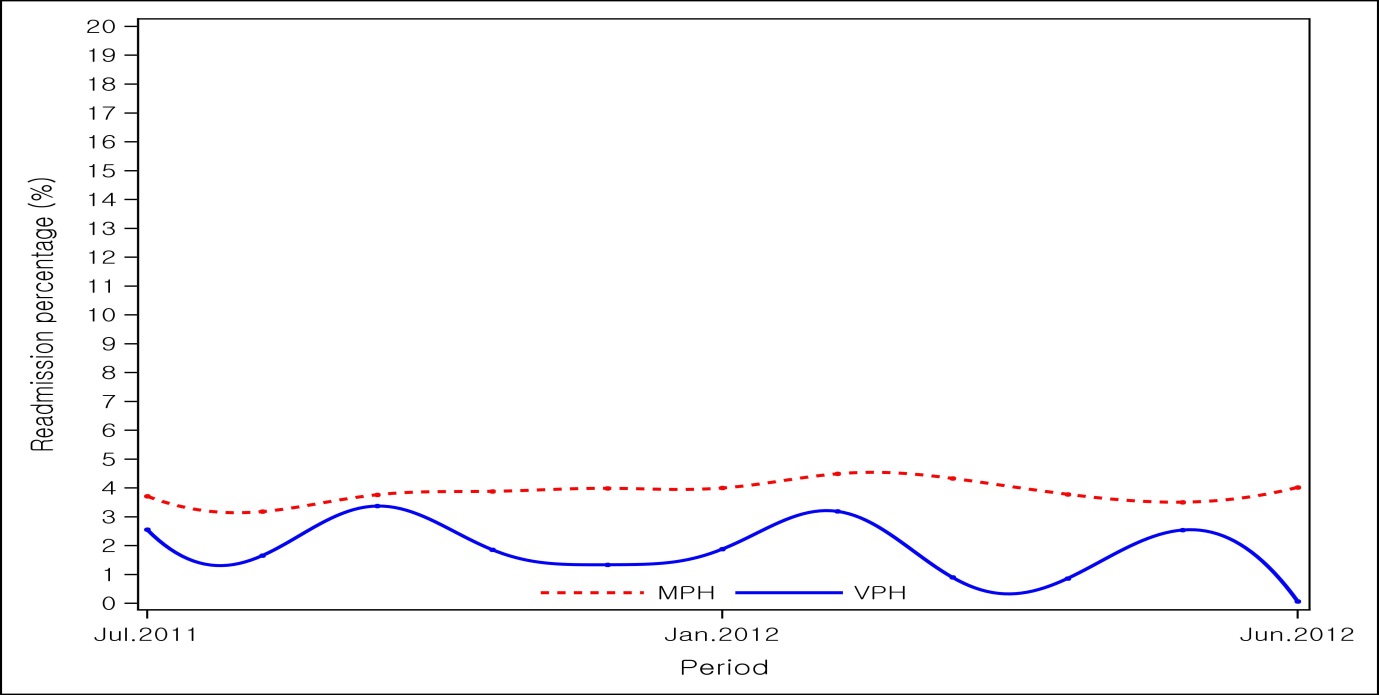 |
| (C) shifting services to outpatient settings | (D) Readmission |

Figure S1. Parallel trend assumption in case of large hospital

| 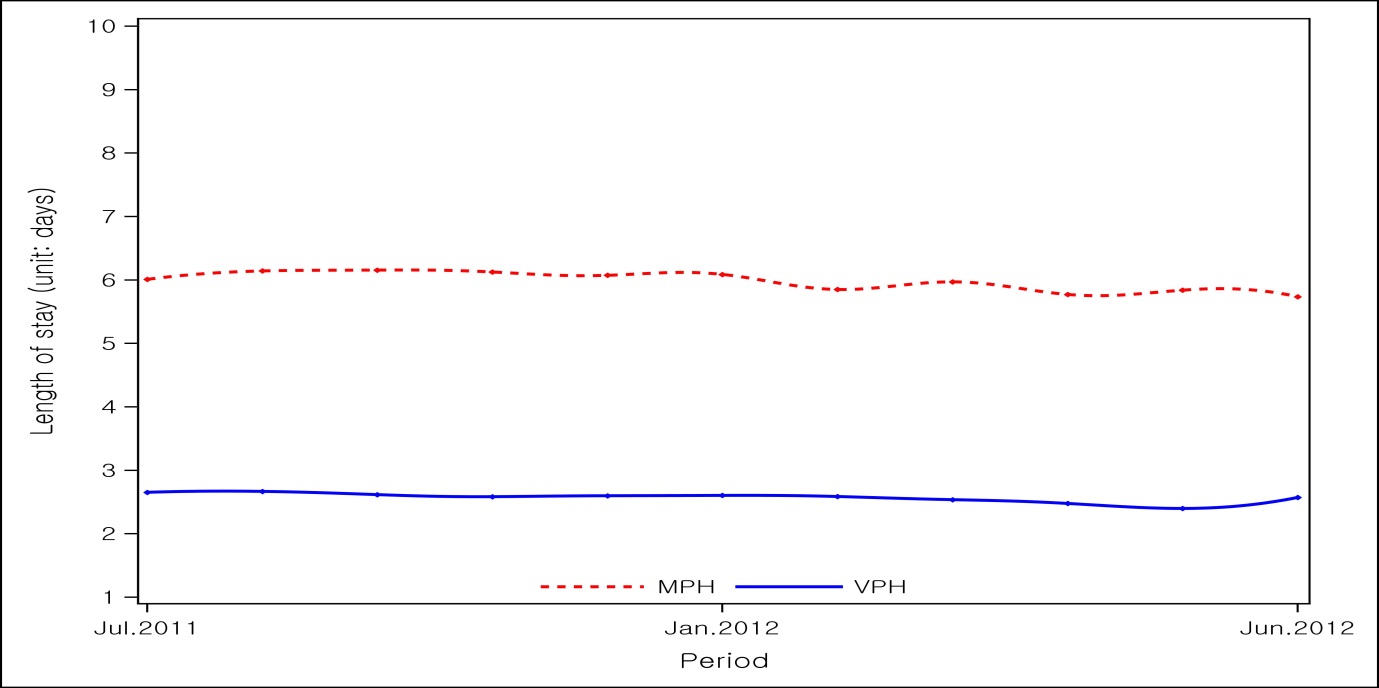 | 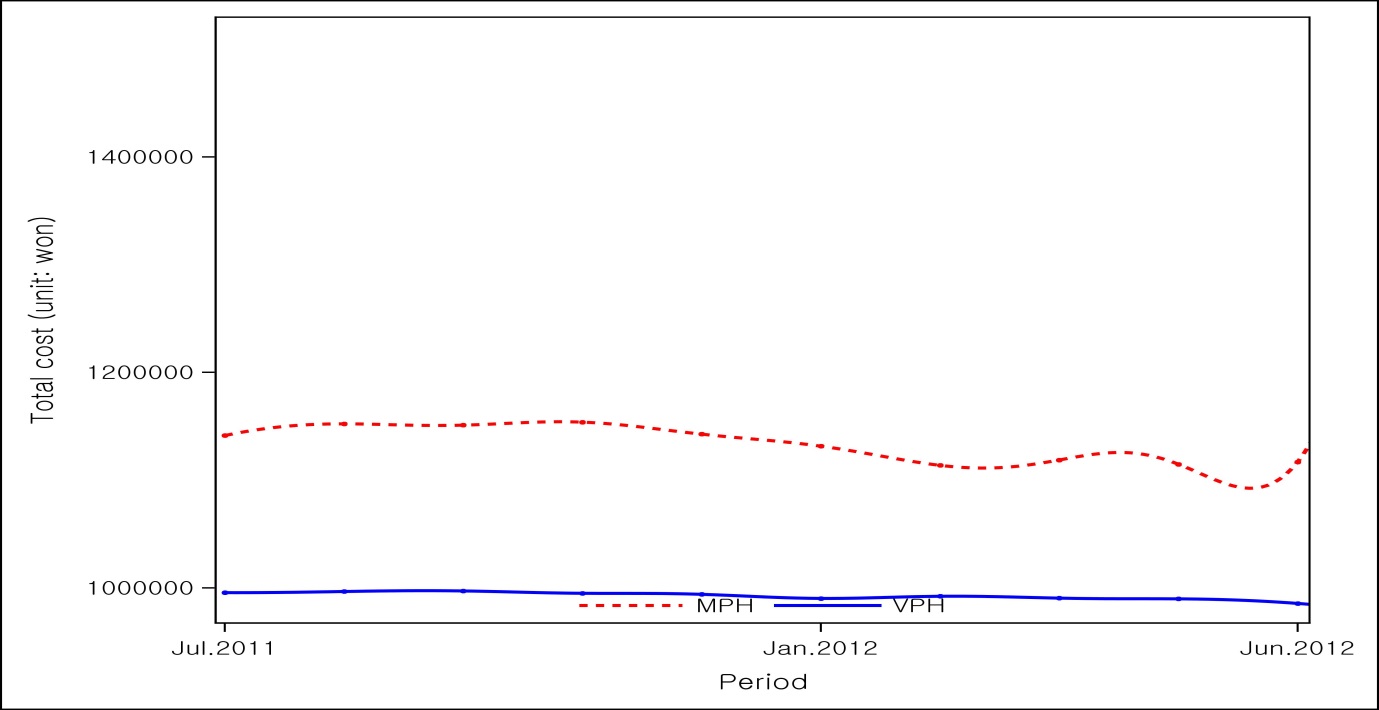 |
| --- | --- |
| (A) Length-of-stay | (B) Total medical costs |
| 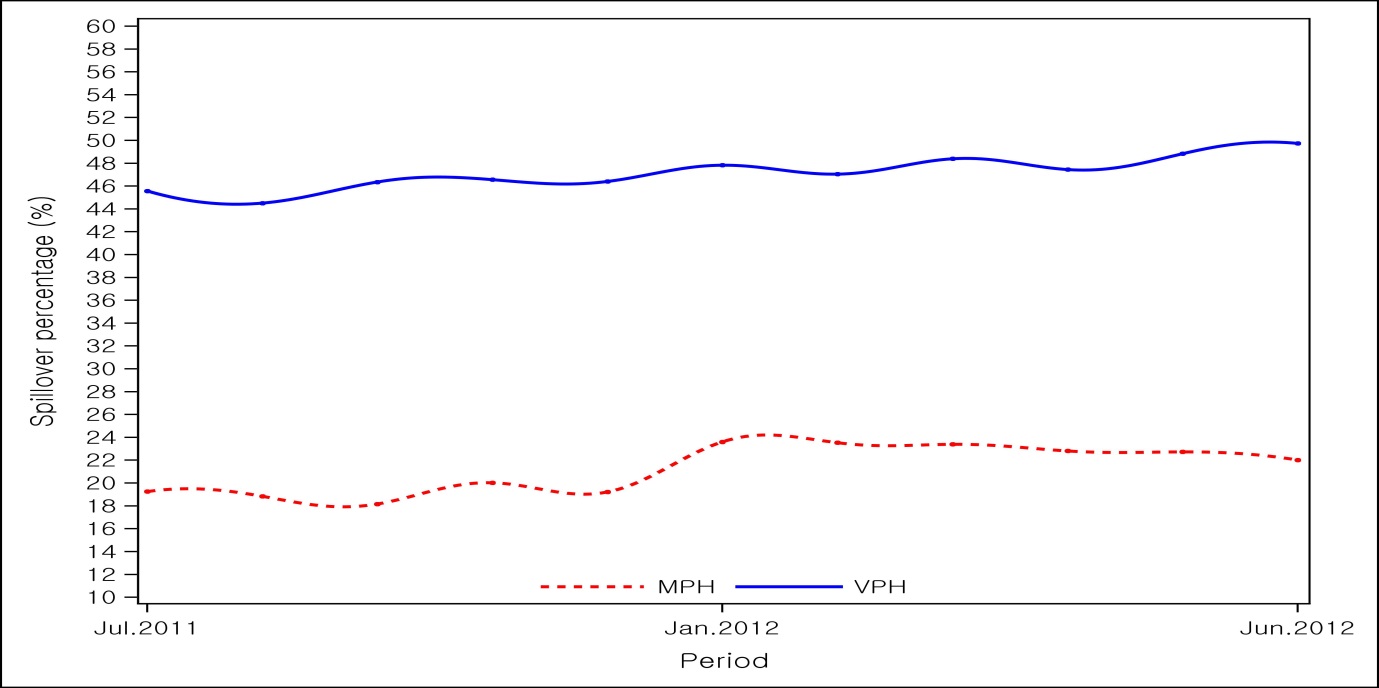 | 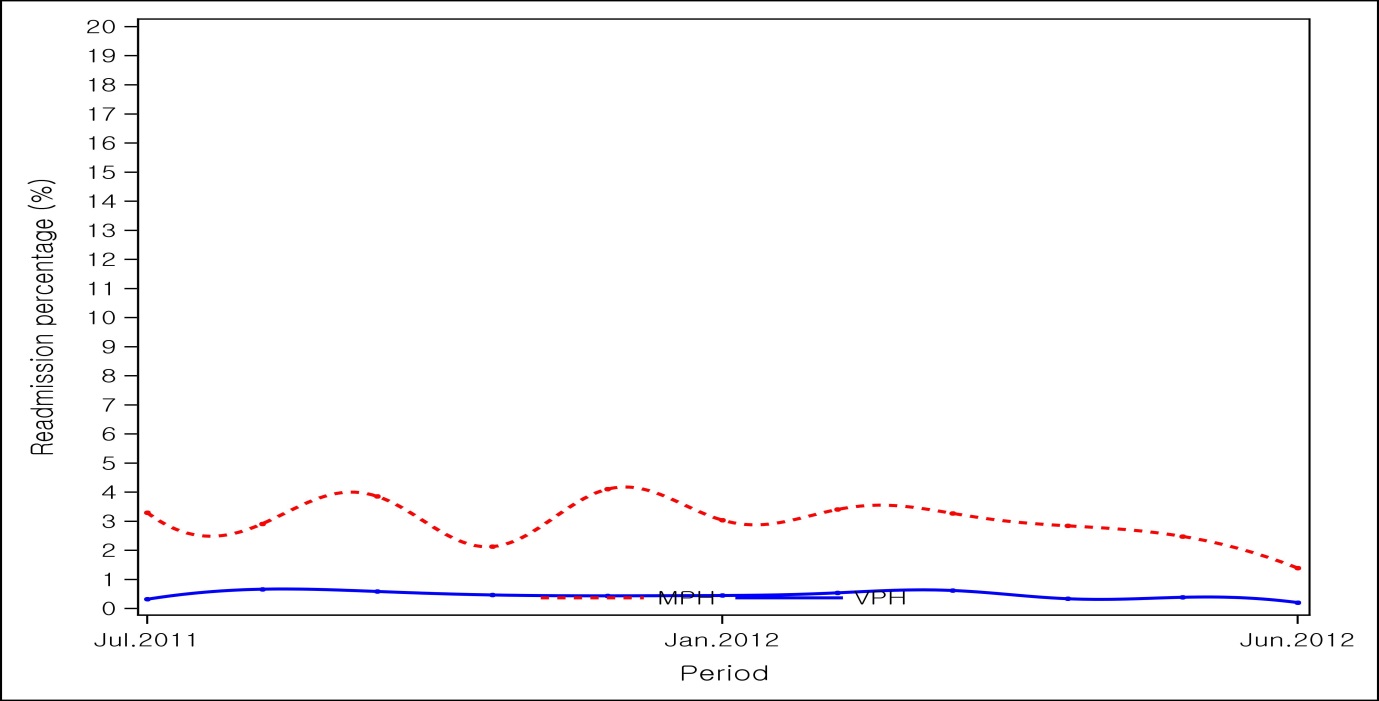 |
| (C) shifting services to outpatient settings | (D) Readmission |

Figure S2. Parallel trend assumption in case of small hospital
