# Supplementary material for: Effects of a mandatory DRG payment system in South Korea: Analysis of multi-year nationwide hospital claims data
Source: BMC Health Serv Res. 2019 Oct 30;19:776. doi: 10.1186/s12913-019-4650-8 (PMC6822472; doi:10.1186/s12913-019-4650-8)
Supplement: Supplementary file 2 — Additional file 2: Table S1. Regression model estimates for length-of-stay according to DRG policy intervention period and primary diagnosis. Table S2. Regression model estimates for total medical cost according to DRG policy intervention period and primary diagnosis. Table S3. Regression model estimates for shifting services to outpatient settings according to DRG policy intervention and primary diagnosis. Table S4. Regression model estimates for readmission according to DRG policy intervention and primary diagnosis. [file 12913_2019_4650_MOESM2_ESM.doc]

| Table S1. Regression model estimates for length-of-stay according to DRG policy intervention period and primary diagnosis | | | | | | | | |
| --- | --- | --- | --- | --- | --- | --- | --- | --- |
| Variables | | Pre-Intervention | First Intervention | | | Second Intervention | | |
|  |  | 7/2011-6/2012 | 7/2012-6/2013 | | | 7/2013~6/2014 | | |
|  |  | Mean±SD | Mean±SD | *B* | DID | Mean±SD | *B* | DID |
| Cataract surgery |  |  |  |  |  |  |  |  |
| Large hospital | MPH | 1.72±1.63 | 1.68±1.53 | 0.98*** | 1.02 | 1.34±0.84 | 0.86*** | 0.89*** |
|  | VPH | 2.12±1.31 | 2.03±1.23 | 0.96*** |  | 1.97±1.17 | 0.96*** |  |
| Small hospital | MPH | 1.26±1.11 | 1.01±0.19 | 0.97 | 1.11*** | 1.01±0.13 | 1.00 | 1.01*** |
|  | VPH | 1.03±0.23 | 1.03±0.20 | 1.00*** |  | 1.02±0.18 | 1.00*** |  |
| Tonsillectomy & adenoidectomy | |  |  |  |  |  |  |  |
| Large hospital | MPH | 4.84±2.84 | 4.69±2.67 | 0.97*** | 1.09*** | 3.46±1.29 | 0.80*** | 0.84*** |
|  | VPH | 4.67±2.80 | 4.04±2.63 | 0.91*** |  | 3.56±1.24 | 0.95*** |  |
| Small hospital | MPH | 3.18±2.58 | 2.75±1.70 | 0.89*** | 0.86*** | 2.60±1.65 | 0.95*** | 0.86*** |
|  | VPH | 2.62±2.17 | 2.68±1.71 | 1.02 |  | 3.00±2.00 | 1.06** |  |
| Appendectomy |  |  |  |  |  |  |  |  |
| General hospital | MPH | 6.12±3.09 | 5.98±2.95 | 0.98*** | 1.00 | 5.11±2.22 | 0.88*** | 0.91*** |
|  | VPH | 6.15±2.95 | 6.01±2.78 | 0.98** |  | 5.69±2.35 | 0.96*** |  |
| Hospital | MPH | 6.49±2.99 | 5.60±2.38 | 0.88*** | 0.92*** | 5.27±2.21 | 0.94*** | 0.96*** |
|  | VPH | 5.60±2.57 | 5.31±2.35 | 0.96*** |  | 5.26±2.42 | 0.99* |  |
| Herniotomy |  |  |  |  |  |  |  |  |
| Large hospital | MPH | 4.76±2.68 | 4.70±2.74 | 0.98*** | 1.01 | 3.83±1.66 | 0.85*** | 0.89*** |
|  | VPH | 5.22±2.65 | 5.05±2.55 | 0.97** |  | 4.75±2.20 | 0.95*** |  |
| Small hospital | MPH | 5.75±3.36 | 4.60±2.34 | 0.84*** | 1.00 | 4.23±2.10 | 0.95*** | 0.95** |
|  | VPH | 3.51±1.85 | 3.04±1.74 | 0.86*** |  | 2.99±1.64 | 0.98* |  |
| Hemorrhoidectomy |  |  |  |  |  |  |  |  |
| Large hospital | MPH | 5.55±4.31 | 5.52±4.42 | 0.98** | 1.00 | 3.93±2.05 | 0.78*** | 0.80*** |
|  | VPH | 4.16±2.76 | 4.08±2.62 | 0.98* |  | 3.86±2.08 | 0.97*** |  |
| Small hospital | MPH | 4.89±4.40 | 3.31±2.12 | 0.81*** | 0.88*** | 2.92±1.72 | 0.95*** | 1.01 |
|  | VPH | 2.96±1.47 | 2.82±1.39 | 0.95*** |  | 2.75±1.36 | 0.97*** |  |
| Hysterectomy |  |  |  |  |  |  |  |  |
| Large hospital | MPH | 6.63±3.00 | 6.57±3.05 | 0.98*** | 1.00 | 5.59±2.06 | 0.88*** | 0.91*** |
|  | VPH | 6.36±2.38 | 6.23±2.30 | 0.98*** |  | 6.01±2.19 | 0.97*** |  |
| Small hospital | MPH | 6.67±2.47 | 5.80±2.43 | 0.85*** | 0.88*** | 5.30±2.32 | 0.90*** | 0.94*** |
|  | VPH | 5.43±2.19 | 5.33±2.22 | 0.96*** |  | 5.13±2.14 | 0.96*** |  |
| Cesarean delivery |  |  |  |  |  |  |  |  |
| Large hospital | MPH | 8.10±6.58 | 7.84±5.99 | 0.98*** | 1.01 | 6.14±1.64 | 0.86*** | 0.88*** |
|  | VPH | 7.57±3.78 | 7.12±1.67 | 0.97*** |  | 6.88±1.17 | 0.97*** |  |
| Small hospital | MPH | 7.03±2.42 | 6.71±1.11 | 0.97*** | 0.97*** | 6.58±1.11 | 0.99*** | 0.98*** |
|  | VPH | 6.49±1.70 | 6.42±1.12 | 0.99*** |  | 6.42±1.12 | 1.00*** |  |

MPH: mandatory participation hospitals

VPH: voluntary participation hospitals

DID: difference-in-differences

Large hospital: tertiary hospitals + general hospitals; Small hospital: hospitals+ clinics.

Adjusted odds ratios obtained from multiple regression analysis with all of the variables in Table 1.

*p<0.05 **p<0.01 ***p<0.001

| Table S2. Regression model estimates for total medical cost according to DRG policy intervention period and primary diagnosis | | | | | | | | |
| --- | --- | --- | --- | --- | --- | --- | --- | --- |
| Variables | | Pre-Intervention | First Intervention | | | Second Intervention | | |
|  |  | 7/2011-6/2012 | 7/2012-6/2013 | | | 7/2013~6/2014 | | |
|  |  | Mean±SD | Mean±SD | *B* | DID | Mean±SD | *B* | DID |
| Cataract surgery |  |  |  |  |  |  |  |  |
| Large hospital | MPH | 808±324 | 766±309 | 0.95*** | 1.03*** | 823±174 | 1.11*** | 1.08*** |
|  | VPH | 978±344 | 872±273 | 0.92*** |  | 896±260 | 1.01*** |  |
| Small hospital | MPH | 690±162 | 687±46 | 1.05*** | 1.24*** | 681±37 | 0.99*** | 1.00 |
|  | VPH | 818±46 | 688±44 | 0.84*** |  | 681±39 | 0.99*** |  |
| Tonsillectomy & adenoidectomy | |  |  |  |  |  |  |  |
| Large hospital | MPH | 795±402 | 764±337 | 0.97*** | 1.07*** | 730±70 | 1.03*** | 1.05*** |
|  | VPH | 765±265 | 748±206 | 1.02 |  | 719±51 | 0.99* |  |
| Small hospital | MPH | 541±251 | 604±73 | 1.21*** | 0.93*** | 595±68 | 0.99*** | 0.98*** |
|  | VPH | 494±174 | 601±79 | 1.29*** |  | 612±110 | 1.00 |  |
| Appendectomy |  |  |  |  |  |  |  |  |
| Large hospital | MPH | 1873±593 | 1860±557 | 0.99*** | 0.89*** | 2049±360 | 1.13*** | 1.10*** |
|  | VPH | 1739±467 | 1943±412 | 1.12*** |  | 1969±320 | 1.02*** |  |
| Small hospital | MPH | 1540±411 | 1777±353 | 1.17*** | 1.02*** | 1787±312 | 1.01*** | 1.02*** |
|  | VPH | 1472±334 | 1700±344 | 1.15*** |  | 1704±327 | 1.00 |  |
| Herniotomy |  |  |  |  |  |  |  |  |
| Large hospital | MPH | 1183±463 | 1170±458 | 0.99** | 1.02* | 1233±352 | 1.08*** | 1.05*** |
|  | VPH | 1131±381 | 1078±257 | 0.97*** |  | 1107±257 | 1.02*** |  |
| Small hospital | MPH | 1063±388 | 1010±244 | 0.99 | 0.99 | 1035±259 | 1.03*** | 1.02* |
|  | VPH | 905±154 | 913±207 | 1.00 |  | 932±227 | 1.01*** |  |
| Hemorrhoidectomy |  |  |  |  |  |  |  |  |
| Large hospital | MPH | 983±616 | 975±637 | 0.99* | 0.90*** | 871±205 | 0.98*** | 0.98** |
|  | VPH | 884±267 | 974±256 | 1.10*** |  | 963±209 | 1.00 |  |
| Small hospital | MPH | 789±351 | 737±185 | 1.04*** | 1.01*** | 719±162 | 1.00 | 1.01*** |
|  | VPH | 692±125 | 704±130 | 1.02*** |  | 701±136 | 0.99*** |  |
| Hysterectomy |  |  |  |  |  |  |  |  |
| Large hospital | MPH | 1670±604 | 1643±600 | 0.98*** | 0.81*** | 2170±431 | 1.38*** | 1.26*** |
|  | VPH | 1661±372 | 2002±398 | 1.22*** |  | 2180±405 | 1.09*** |  |
| Small hospital | MPH | 1261±437 | 1753±389 | 1.47*** | 1.20*** | 1853±403 | 1.05*** | 1.01* |
|  | VPH | 1430±345 | 1745±393 | 1.22*** |  | 1828±423 | 1.04*** |  |
| Cesarean delivery |  |  |  |  |  |  |  |  |
| Large hospital | MPH | 1388±847 | 1367±845 | 0.99*** | 0.86*** | 1623±381 | 1.28*** | 1.33*** |
|  | VPH | 1369±376 | 1568±312 | 1.15*** |  | 1426±198 | 0.96*** |  |
| Small hospital | MPH | 939±243 | 1347±182 | 1.46 | 1.23*** | 1361±159 | 1.01*** | 0.99*** |
|  | VPH | 1095±166 | 1330±174 | 1.18*** |  | 1351±156 | 1.02*** |  |

MPH: mandatory participation hospitals

VPH: voluntary participation hospitals

DID: difference-in-differences

Large hospital: tertiary hospitals + general hospitals; Small hospital: hospitals+ clinics.

Adjusted odds ratios obtained from multiple regression analysis with all of the variables in Table 1.

All costs were controlled by medical insurance fees, which are annually adjusted and include un-insured costs.

1 USD = 1,200 Korean won (02/2016)

*p<0.05 **p<0.01 ***p<0.001

| Table S3. Regression model estimates for shifting services to outpatient settings according to DRG policy intervention and primary diagnosis | | | | | | | | |
| --- | --- | --- | --- | --- | --- | --- | --- | --- |
| Variables | | Pre-Intervention | First Intervention | | | Second Intervention | | |
|  |  | 7/2011-6/2012 | 7/2012-6/2013 | | | 7/2013~6/2014 | | |
|  |  | N (%) | N (%) | *B* | DID | Mean±SD | *B* | DID |
| Cataract surgery |  |  |  |  |  |  |  |  |
| Large hospital | MPH | 15131(39.00) | 18358(45.11) | 1.28*** | 0.91* | 20649(46.76) | 1.07*** | 1.15** |
|  | VPH | 2173(45.87) | 2377(54.34) | 1.46*** |  | 2236(52.41) | 0.92 |  |
| Small hospital | MPH | 117(42.09) | 3469(33.11) | 1.14 | 2.63*** | 9279(30.99) | 0.84*** | 0.97 |
|  | VPH | 174115(50.71) | 168145(49.57) | 0.96*** |  | 162189(48.97) | 0.97*** |  |
| Tonsillectomy & adenoidectomy | | |  |  |  |  |  |  |
| Large hospital | MPH | 3486(47.64) | 3944(54.38) | 1.31*** | 0.81 | 3803(60.97) | 1.26*** | 1.14 |
|  | VPH | 315(45.19) | 323(57.58) | 1.66*** |  | 316(60.31) | 1.20*** |  |
| Small hospital | MPH | 1534(54.75) | 1567(52.67) | 0.96 | 0.73** | 1402(47.87) | 0.82*** | 0.63*** |
|  | VPH | 709(53.79) | 468(60.00) | 1.26* |  | 484(67.79) | 1.53*** |  |
| Appendectomy |  |  |  |  |  |  |  |  |
| Large hospital | MPH | 4724(12.04) | 6274(15.41) | 1.32*** | 0.91 | 6807(16.91) | 1.13*** | 1.22*** |
|  | VPH | 712(9.82) | 844(13.85) | 1.48*** |  | 736(13.11) | 0.94 |  |
| Small hospital | MPH | 980(8.68) | 1330(12.04) | 1.42*** | 1.23** | 1343(12.54) | 1.03 | 0.93 |
|  | VPH | 852(12.51) | 841(14.38) | 1.15** |  | 770(15.68) | 1.11 |  |
| Herniotomy |  |  |  |  |  |  |  |  |
| Large hospital | MPH | 4761(42.89) | 5530(47.25) | 1.22*** | 0.97 | 6066(48.91) | 1.08** | 1.11 |
|  | VPH | 788(34.38) | 793(39.97) | 1.25*** |  | 758(39.73) | 0.98 |  |
| Small hospital | MPH | 494(29.11) | 674(35.29) | 1.29*** | 1.15 | 817(38.02) | 1.12*** | 1.14 |
|  | VPH | 1813(36.69) | 2011(39.64) | 1.15*** |  | 1895(38.82) | 0.96 |  |
| Hemorrhoidectomy |  |  |  |  |  |  |  |  |
| Large hospital | MPH | 4754(40.56) | 5934(48.10) | 1.38*** | 0.86*** | 6400(51.11) | 1.11*** | 1.12** |
|  | VPH | 3463(44.09) | 3812(55.75) | 1.60*** |  | 3660(55.59) | 1.00 |  |
| Small hospital | MPH | 4287(43.38) | 7483(51.75) | 1.22*** | 1.08** | 12028(59.21) | 1.29*** | 1.31*** |
|  | VPH | 153564(63.83) | 157009(67.51) | 1.18*** |  | 147365(67.61) | 1.01 |  |
| Hysterectomy |  |  |  |  |  |  |  |  |
| Large hospital | MPH | 20920(42.70) | 22230(46.07) | 1.15*** | 1.03 | 22229(47.31) | 1.05*** | 1.01 |
|  | VPH | 5288(38.43) | 5170(41.17) | 1.12*** |  | 5129(42.22) | 1.04 |  |
| Small hospital | MPH | 3313(30.05) | 4168(32.78) | 1.15*** | 0.99 | 4764(32.19) | 0.98 | 0.94 |
|  | VPH | 6918(33.29) | 7188(37.04) | 1.16*** |  | 6930(37.83) | 1.03 |  |
| Cesarean delivery |  |  |  |  |  |  |  |  |
| Large hospital | MPH | 1612(7.75) | 2128(10.23) | 1.37*** | 0.97 | 2406(12.20) | 1.24*** | 0.57*** |
|  | VPH | 634(6.31) | 792(8.53) | 1.47*** |  | 1412(16.72) | 2.26*** |  |
| Small hospital | MPH | 1416(2.95) | 1356(2.46) | 0.89*** | 0.76*** | 1920(3.17) | 1.35*** | 1.10* |
|  | VPH | 1608(1.89) | 1682(2.11) | 1.12*** |  | 1779(2.55) | 1.22*** |  |

MPH: mandatory participation hospitals

VPH: voluntary participation hospitals

DID: difference-in-differences

Large hospital: tertiary hospitals + general hospitals; Small hospital: hospitals+ clinics.

Adjusted odds ratios obtained from multiple regression analysis with all of the variables in Table 1.

*p<0.05 **p<0.01 ***p<0.001

| Table S4. Regression model estimates for readmission according to DRG policy intervention and primary diagnosis | | | | | | | | |
| --- | --- | --- | --- | --- | --- | --- | --- | --- |
| Variables | | Pre-Intervention | First Intervention | | | Second Intervention | | |
|  |  | 7/2011-6/2012 | 7/2012-6/2013 | | | 7/2013~6/2014 | | |
|  |  | N (%) | N (%) | *B* | DID | Mean±SD | *B* | DID |
| Cataract surgery |  |  |  |  |  |  |  |  |
| Large hospital | MPH | 4835(12.46) | 5323(13.08) | 1.07** | 1.21 | 82(0.19) | 0.01*** | 0.11*** |
|  | VPH | 117(2.47) | 99(2.26) | 0.91 |  | 11(0.26) | 0.15*** |  |
| Small hospital | MPH | 62(22.3) | 7(0.07) | 0.00*** | 0.01*** | 8(0.03) | 0.39 | 0.66 |
|  | VPH | 183(0.05) | 140(0.04) | 0.92 |  | 80(0.02) | 0.58*** |  |
| Tonsillectomy & adenoidectomy | |  |  |  |  |  |  |  |
| Large hospital | MPH | 737(10.07) | 676(9.32) | 0.92 | 0.87 | 644(10.32) | 1.14* | 1.13 |
|  | VPH | 58(8.32) | 49(8.73) | 1.03 |  | 46(8.78) | 1.06 |  |
| Small hospital | MPH | 225(8.03) | 180(6.05) | 0.72 | 0.75 | 143(4.88) | 0.82 | 0.72 |
|  | VPH | 75(5.69) | 42(5.38) | 1.00 |  | 44(6.16) | 1.30 |  |
| Appendectomy |  |  |  |  |  |  |  |  |
| Large hospital | MPH | 1083(2.76) | 1159(2.85) | 1.02 | 1.50** | 596(1.48) | 0.52*** | 0.73 |
|  | VPH | 138(1.90) | 79(1.30) | 0.70* |  | 52(0.93) | 0.73 |  |
| Small hospital | MPH | 382(3.39) | 147(1.33) | 0.39*** | 0.48*** | 132(1.23) | 0.92 | 1.02 |
|  | VPH | 116(1.70) | 80(1.37) | 0.80 |  | 61(1.24) | 0.91 |  |
| Herniotomy |  |  |  |  |  |  |  |  |
| Large hospital | MPH | 208(1.87) | 211(1.80) | 0.96 | 2.31* | 145(1.17) | 0.65*** | 0.98 |
|  | VPH | 31(1.35) | 11(0.55) | 0.45* |  | 7(0.37) | 0.69 |  |
| Small hospital | MPH | 47(2.77) | 21(1.10) | 0.40*** | 0.41* | 22(1.02) | 0.88 | 0.75 |
|  | VPH | 32(0.65) | 32(0.63) | 0.98 |  | 37(0.76) | 1.20 |  |
| Hemorrhoidectomy |  |  |  |  |  |  |  |  |
| Large hospital | MPH | 467(3.98) | 467(3.79) | 0.95 | 0.94 | 249(1.99) | 0.53*** | 0.70* |
|  | VPH | 182(2.32) | 160(2.34) | 1.01 |  | 116(1.76) | 0.73* |  |
| Small hospital | MPH | 340(3.44) | 197(1.36) | 0.43*** | 0.47*** | 256(1.26) | 0.95 | 1.05 |
|  | VPH | 2611(1.09) | 2483(1.07) | 0.98 |  | 2250(1.03) | 0.96 |  |
| Hysterectomy |  |  |  |  |  |  |  |  |
| Large hospital | MPH | 1384(2.82) | 1286(2.67) | 0.94 | 1.09 | 653(1.39) | 0.51*** | 0.73* |
|  | VPH | 140(1.02) | 111(0.88) | 0.85 |  | 76(0.63) | 0.71 |  |
| Small hospital | MPH | 188(1.71) | 122(0.96) | 0.56*** | 0.55*** | 141(0.95) | 0.99 | 1.19 |
|  | VPH | 136(0.65) | 130(0.67) | 1.01 |  | 102(0.56) | 0.84 |  |
| Cesarean delivery |  |  |  |  |  |  |  |  |
| Large hospital | MPH | 263(1.26) | 223(1.07) | 0.84 | 1.63 | 25(0.13) | 0.12*** | 0.86 |
|  | VPH | 48(0.48) | 23(0.25) | 0.57** |  | 3(0.04) | 0.14*** |  |
| Small hospital | MPH | 394(0.82) | 35(0.06) | 0.08*** | 0.16*** | 41(0.07) | 1.13 | 1.12 |
|  | VPH | 133(0.16) | 62(0.08) | 0.50*** |  | 54(0.08) | 0.99 |  |

MPH: mandatory participation hospitals

VPH: voluntary participation hospitals

DID: difference-in-differences

Large hospital: tertiary hospitals + general hospitals; Small hospital: hospitals+ clinics.

Adjusted odds ratios obtained from multiple regression analysis with all of the variables in Table 1.

Patients with cataract surgery as the primary diagnosis were excluded in total analysis.

*p<0.05 **p<0.01 ***p<0.001
